# A Dentist Interviewed

**Published:** 1884-09

**Authors:** 


### EDITORIAL, ETC.

A Dentist Interviewed.—An amusing account of an alleged interview between the reporter of a prominent paper and a dentist has recently been published, the non-advertising portion of which we give as follows:

The dentist talked while he worked.

"Of course I study faces," he said, "and I flatter myself that I can read them fairly well, too I am seldom mistaken in my estimate of a patient. Frequently I can tell a man's business before I have him in the chair five minutes. It is one of the delights of my profession. Take your own case, if you'll excuse my personal. Before I had got this tooth half filled I concluded that you are a Southerner and a school teacher. Am I right? No, no; don't move your head? I am seldom wrong. Had a brother a school teacher once. He died. Take the ladies, for instance. I can tell without exchanging a word whether a woman comes here meaning to get the work done at any reasonable cost, or whether she is inclined to higgle over prices. I can guess at a glance whether a patient is going to be troublesome and cross or not. Now I am going to hurt you."

The little wheel whizzed around like a buzz saw, the patient tried in vain to suppress a groan, and the dentist ceased talking. Soon he began again.

"I was going to tell you that the young ladies exhibit more pluck in the chair than the men do. They are not so impatient, for one thing, and they don't like to show any evidence of timidity. Some of them make rather curious requests sometimes, and I have to be very careful about humoring them. I was examining a young lady's teeth yesterday. 'Oh, won't you please hold my hand,' she said, 'it will give me much confidence.' To be sure I didn't do it. I have been in the business long enough to be discreet. Some of these artful creatures seem to do everything in their power to drive a young dentist to distraction. After fighting one blackmail suit in the courts, I am cautious. At a signal from me two ladies will sit on that sofa doing fancy work, and the double doors are arranged so that they can see everything which goes on in this room. After a New York girl has been to a dentist once and got confidence in him, she never thinks of bringing her sister or her mamma with her again. Bashful, timid girls from the country always bring three or four relatives with them, who invariably get impatient and hinder me in my work. Elderly women, especially single ones, are the most suspicious patients to deal with. They want to know the why and wherefore of every detail, and they always affect astonishment at the price. A few days since one of them wanted a set of upper teeth made.

"How much will you charge?" she asked.

"I told her $45."

"Gracious" she said, "I thought I could get top and bottom for $12.

"I thought that she might possibly be in poor circumstances, and did the work for her very cheap. I have since learned that she could buy and sell me out four times over, and that she gave me a fictitious name. Another trouble with the women is in getting them to pay for broken appointments. Any trifling thing, a caller, or an hour's extra sleep, will cause them to miss an engagement I have set aside for them, or come late. Then they make a wretched fuss on being asked to pay for time unnecessarily squandered. Exceptional cases, either bereavement or sickness, are, of course, allowed for."

"Business men are the hardest fellows to deal with. They want us to do everything with a down-town rush. The operation is put off and put off again until a cold snap or a drink of ice water hurries matters a little. I have frequently to make evening and Sunday engagements for rich men who will not spare any other time from their business. They pay extra prices. I had a young broker come in here once to have two back teeth filled, I had hardly begun work when he called for a hand glass."

"Oh, never mind," he said. "I can't wait. Those fellows won't show. Take 'em out."

"I remonstrated, but he insisted. When they were out he was angry. That same man will fritter away a couple of hours over his lunch every day. Another thing, I find that with all patients the surroundings go for a great deal. They like a cheerful place. I had an office once which faced the kitchen of a big hotel The chair must be near the window. All day long the room was filled with the aroma from the flesh-pots and the noisy jargon of the scullions. It made the ladies fretful, and—excuse me.

The dentist answered the bell and his patient lifted his head from the torture rack and looked around the office. Everything had obviously been arranged to please the eye. The operatiug chair was drawn close to a window overlooking a model back yard exuberant with foliage and tragrant with flowers. Canary birds chirped and twittered in their cages, goldfish chased one another among floating vines in a big aquarium. There were no gewgaws stuck around on the shelves. The books on the table and the pictures on the wall had evidently been chosen with care. Almost unconsciously one felt quite at home. The dentist returned and was asked this question:

"Why is it that so many dentists are without the modern appliances for extracting teeth? They seem to have ignored that branch of the business."

The dentist continued his work and his conversation. "Our business is to save teeth, not to pull them out," he said. "It does not pay many of us to bother with extracting. That work seems to have fallen into the hands of dentists who do nothing else. I does not require much knowledge—though sometimes considerable muscle—to pull a tooth. Barbers used to do it. In England to-day people run to apothecaries to have teeth drawn. Dentistry has now become one of the fine arts. If a tooth is too far gone to be filled we clip away the edges and put in a porcelain one on a pivot, so that you could hardly tell the difference. Gold and silver is now made much more suitable for our purpose than it used to be. The gold is of better quality, softer, and more easily worked. A

few years ago 'building' a tooth, as is now commonly practiced, would have been a difficult, and, in some cases, impossible job. Big operations bring big prices. Some New York dentists get apparently fabulous prices for their work, but I suppose skilled labor is worth what it will bring in the market. There are dentists who will easily average $40,000 a year. The majority of them, though, earn anywhere from $2,000 to $7,000 a year. Of course I am speaking only of graduates of some dental college and members of one of the local associations. I do not include certificate dentists."

"What do you mean by certificate dentists?"

"Well, quacks. In 1879 a law was passed requiring all dentists who should hereafter be admitted to practice to be regular graduates of some dental college. But all non-graduates who were at that time practicing under certificates were allowed to continue. You will see them scattered all through the city doing work at cut-throat rates. The regulars, of course, don't recognize them, and personally I have no knowledge how much they earn."

"How is a poor mortal suffering with the toothache to distinguish a graduate from a certificate man?"

"Easily enough. The college men are entitled to prefix 'Dr.' to their names. The other fellows are not. It is simply 'John Smith, Dentist."

